# A review of fake news detection approaches: A critical analysis of relevant studies and highlighting key challenges associated with the dataset, feature representation, and data fusion

**DOI:** 10.1016/j.heliyon.2023.e20382

**Published:** 2023-09-21

**Authors:** Suhaib Kh Hamed, Mohd Juzaiddin Ab Aziz, Mohd Ridzwan Yaakub

**Affiliations:** a Center for Software Technology and Management (SOFTAM), Faculty of Information Science and Technology, Universiti Kebangsaan Malaysia (UKM), Bangi 43600, Selangor, Malaysia; b Center for Artificial Intelligence Technology (CAIT), Faculty of Information Science and Technology, Universiti Kebangsaan Malaysia (UKM), Bangi 43600, Selangor, Malaysia

**Keywords:** Fake news detection, Dataset, Overfitting/underfitting, Image feature, Feature vector, Data fusion

## Abstract

Currently, social networks have become the main source to acquire news about current global affairs. However, fake news appears and spreads on social media daily. This disinformation has a negative influence on several domains, such as politics, the economy, and health. In addition, it further generates detriments to societal stability. Several studies have provided effective models for detecting fake news in social networks through a variety of methods; however, there are limitations. Furthermore, since it is a critical field, the accuracy of the detection models was found to be notably insufficient. Although many review articles have addressed the repercussions of fake news, most have focused on specific and recurring aspects of fake news detection models. For example, the majority of reviews have primarily focused on dividing datasets, features, and classifiers used in this field by type. The limitations of the datasets, their features, how these features are fused, and the impact of all these factors on detection models were not investigated, especially since most detection models were based on a supervised learning approach. This review article analyzes relevant studies for the few last years and highlights the challenges faced by fake news detection models and their impact on their performance. The investigation of fake news detection studies relied on the following aspects and their impact on detection accuracy, namely datasets, overfitting/underfitting, image-based features, feature vector representation, machine learning models, and data fusion. Based on the analysis of relevant studies, the review showed that these issues significantly affect the performance and accuracy of detection models. This review aims to provide room for other researchers in the future to improve fake news detection models.

## Highlights

• Many fake news detection models were overfitted, underfitted, or misclassified.
• Social media contains slang or mistakes which lead to OOV during embedding.
• Visual features were not widely used in fake news detection.
• Poor feature vector representation led to poor fake news accuracy.
• Multimodal models that simply combine different features lose out on many attributes.

## 1 Introduction

**Fig. 1:**
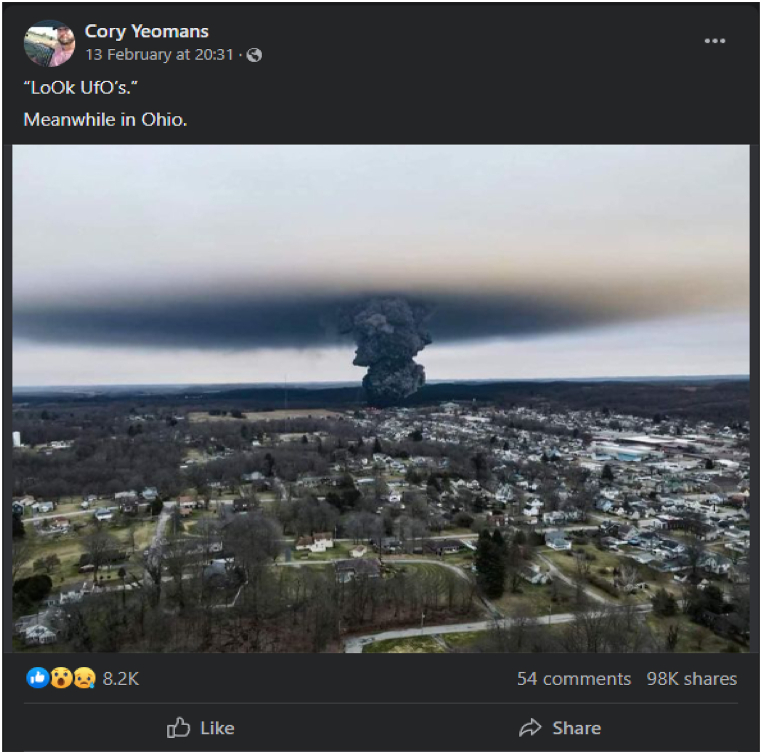
An example of fake news posted on Facebook (https://www.facebook.com/cory.yeomans.1/posts/pfbid0oAw.

The notable proliferation of fake news and its distortion of equity, democracy, and destabilization of public trust has necessitated the need for fake news detection models. Fake news has compromised media trust, leaving readers perplexed. Based on the analysis of the literature, it appears that fake news has been responsible for numerous real-time disasters and is detrimental to the economy [1], health [2], political stability [3], and journalism in general [4]. Manual interferences are ineffective at curbing fake news dissemination due to high-speed data distribution [5]. Currently, many people across the globe rely on social media for world news. In contrast to traditional media, social networks provide quick, free, and unrestricted propagation of posts to a large audience in a short amount of time [6]. Fig. 1 shows the large number of comments and shares on fake news posted on Facebook.

FDWuxojqYHV4TZQBKsPD86mtr3bx38J64ffEMvbGYWdJeuGFwcMDdMxdhx18Al, accessed on May 16, 2023)

Social networks have several advantages along with disadvantages, thereby contributing to the spread of fake news on social media [7]. These platforms may be exploited to spread false information for malicious ends [8], such as inciting hatred based on extremist ideologies, influencing public opinion for political purposes, forming biased opinions to win elections, or financial gain [9,10]. With respect to the health aspect, fake news, and misinformation were found to spread widely on social media following the COVID-19 pandemic outbreak. Misinformation regarding COVID-19 spread faster than real news. In these circumstances, public mental health was compromised, and disease anxiety was widespread [11]. In April 2020, more than 4000 fake news articles included misinformation regarding the COVID-19 epidemic, increasing fear amongst the already panic-stricken public. In addition, some of the fake news prescribed inaccurate treatments and practices that may have led been fatal or worsening of health. In February 2020, at the Security Council meeting held in Munich, it was indicated that the world needed to confront the epidemic as well as the infodemic [2]. In a related matter, Facebook deleted more than 12 million posts containing false information about COVID-19 and vaccines [12]. These negative aspects of social media, represented by the dissemination of fake news, portend a serious threat that has a negative impact on society and citizens’ daily lives. Social network sites and web-based forums have caused fake news sharing a primary issue for various agencies and organizations. Detecting fake news is one of the current topics of research in academia, as it can be investigated from various disciplinary angles [13]. Although studies discussing this issue and providing solutions to curb fake news are still in their early stages, they are steadily increasing. However, this requires exploring various directions in research along with further development of fake news detection models [4,6]. Previous research has successfully attempted to identify fake news in social networks through diverse methods; nonetheless, they still face certain limitations. Moreover, the precision of detection models has been found to be notably insufficient (e.g., the detection rate was low while the processing duration for detection was significant). Despite the abundance of research in this arena, most previous reviews have focused on specific and repeated aspects of fake news detection models. For example, many review articles only reviewed datasets used in this field. Some of these reviews divided the datasets based on content, domain, labels, etc. The limitations of these datasets and their impact on detection models were not investigated, especially since most detection models were based on a supervised learning approach. Another problem associated with the dataset that results from the use of a small or imbalanced dataset is misclassification, which causes the detection model to become overfitted or underfitted. In addition, the effectiveness of the use of visual features in detecting fake news not highlighted in previous review articles remains unknown. Another issue that has not received focus in previous reviews is whether a poor representation of the features used might result in poor detection accuracy. Moreover, the limitations surrounding machine learning techniques for detecting fake news also need to be addressed. Finally, the issue of the disadvantages of directly fusing different features from different modalities has not been considered in previous review articles in detail. In addition, the review articles failed to address several significant challenges that continue to plague fake news detection models. Consequently, the purpose of this review is to create a platform for future researchers to address these issues and enhance the effectiveness of fake news detection models. The main contributions of this review are as follows.

• Categorizing fake news detection models based on the main approaches.
• Providing a critical analysis of relevant recent studies.
• Highlighting the challenges faced by fake news detection models and affecting their performance accuracy, including (Dataset, overfitting/underfitting, image-based features, feature vector representation, machine learning models, and data fusion).

The remainder of this review is organized as follows: Section 2 summarizes relevant studies that discuss specific aspects of fake news detection. Section 3 investigates the most commonly used approaches in detecting fake news. Section 4 provides a critical analysis of relevant studies and identifies the limitations of these studies. Section 5 highlights the current challenges of fake news detection models. Section 6 proposes future directions for fake news detection. Finally, Section 7 presents the conclusion of this review.

## 2 Related work

Fake news detection has been addressed by several studies in the literature. Some of these studies are related to a specific discipline, while others are classified according to a specific approach. Furthermore, a few studies investigated certain aspects of detecting fake news. This section contains review articles on fake news detection that included one or more of the following issues affecting detection models' performance: Dataset, overfitting/underfitting, image-based features, feature vector representation, machine learning models, and data fusion. Zhou and Zafarani [14] presented a review investigating the methods used to identify fake news from four aspects: false knowledge, style of writing, patterns of spread, and credibility of the source. The authors also shed light on the basic theories associated with the spread of fake news from various disciplines. It was followed by Tanwar and Sharma [15] who published a short review that mentioned the types of fake news and the datasets used. Alam, Cresci [16] reviewed disinformation detection studies and divided them according to content features (image, speech, video, networked, and temporal information) and depicted some of the challenges in employing multimodal detection models. Another article by D'Ulizia, Caschera [17] presented a comprehensive and in-depth review of datasets related to fake news detection. However, Ansar and Goswami [18] presented an extensive review by characterizing fake news identification from a data science perspective, explaining the types of fake news, investigating the types of features used in detection models, and reviewing the most significant standard datasets available in this field. They also highlighted studies of detecting COVID-19 disinformation as a case study. Furthermore, Li and Lei [13] published a review article that categorized studies related to fake news detection by relying on Deep Learning (DL) on the basis of the data structures employed in news classification (text classification, graph classification, and hybrid classification), since they believed that the issue of detecting fake news is a classification problem. Meanwhile, Swapna and Soniya [19] presented a review of fake news detection studies by presenting a classification of features used in fake news detection models, such as linguistic and semantic features, style-based features, and visual features. In the same context, a comprehensive review of multimodal fake news detection studies based on deep learning techniques was published by Hangloo and Arora [20]. In this review, deep learning techniques used in detection models were discussed along with pre-trained models by using transfer learning methods. Moreover, they shed light on the data collection process used to detect fake news and the most relevant standard datasets in this field. In this study, some of the issues related to fake news detection models were briefly mentioned. In the review article by Hu, Wei [21] a comprehensive review of deep learning-based fake news detection methods was presented, which considered different features such as content, social context, and external knowledge. Some of the commonly used datasets and the results of relevant studies were mentioned in their review, and a number of promising directions for the future were provided. In another context, Capuano, Fenza [22] presented a systematic review that included a taxonomy of fake news studies based on machine learning and deep learning models. They also categorized these studies according to pre-trained models for feature extraction and textual content features for detecting fake news and compared the models and features based on the results obtained from the datasets. Table 1 compares the current study with the related review articles mentioned above in terms of analyzing or discussing significant aspects of fake news detection models.

**Table 1:** A relative comparison of this review with various related reviews according to the investigation of certain aspects.

Reference	Analysis of fake news detection studies	Issues associated with the fake news detection model's performance					
		Datasets	Overfitting and underfitting	Image-based features	Feature representation	Machine learning models	Data fusion
Zhou and Zafarani [14]				✓		✓	
Tanwar and Sharma [15]		✓		✓			
Alam, Cresci [16]		✓		✓			✓
D'Ulizia, Caschera [17]		✓					
Ansar and Goswami [18]		✓		✓	✓		
Li and Lei [13]						✓	✓
Swapna and Soniya [19]				✓		✓	
Hangloo and Arora [20]		✓		✓	✓		✓
Hu, Wei [21]		✓		✓	✓		✓
Capuano, Fenza [22]		✓			✓	✓	
Our review	✓	✓	✓	✓	✓	✓	✓

## 3 Fake news detection approaches

**Fig. 2:**
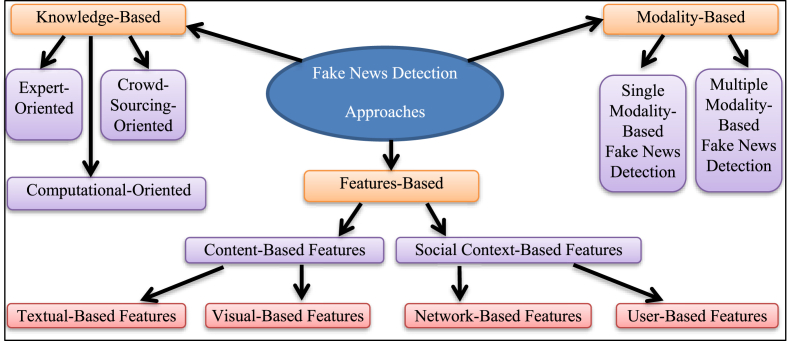
The taxonomy for fake news detection approaches.

The tremendous technological advancement around the globe in recent years, particularly in the mobile phone industry, has made social media platforms accessible and a vital part of our everyday lives [1,23]. Social media supports multimedia posts, including images and videos. Users find visual materials more appealing than text-based information as they can more precisely convey the intended occurrences [24]. In this section, the types of basic approaches used to detect fake news on social media are listed and the way in which the various methods relate to each other for detecting fake news has been discussed. Fake news detection models can be divided based on the following approaches: Knowledge-based approaches, Features-based approaches, and Modality-based approaches [19,25,26], as shown in Fig. 2.

### 3.1 Knowledge-based approaches

From a knowledge standpoint, fact-checking is a procedure used to identify fake news. An independent fact-checker examines news articles and gives actual value to the claims. The three types of fact-checking include expert-oriented, crowd-sourcing-oriented, and computational-oriented [25]. Fact-checking web pages are one of the most reliable sources for creating fake news datasets.

• The expert-oriented method assesses the veracity of the facts by relying on domain-matter experts who analyze data and documents and draw findings. The most popular websites for this task include PolitiFact (politifact.com) and Snopes (snopes.com). Expert-oriented tasks are particularly demanding, as whenever they get an incoming claim, they must contact domain experts and other relevant sources. Since the process is time-consuming, it is not particularly useful [27].
• The crowd-sourced method allows users to have the option to discuss and remark on the veracity of specific news material. It is subject to the expertise of a team of fact-checkers. Due to the political bias and competing interests of fact-checkers, this method, when compared to expert-based fact-checking, is more challenging to manage, less trustworthy, and less accurate [14]. An example of this strategy is Fiskkit (fiskkit.com).
• The computational-oriented approach provides users with an automatic system that categorizes a news item as having true or false material. The applications that focus on computation include structured knowledge graphs and open web sources [19].

### 3.2 Features-based approaches

AI-based models for identifying fake news rely on a number of key features, such as content-based, network-based, or user-based features. However, using all of these features may not improve the performance of the news classifier. The utilization of one or more of these features is based on the type of fake news detection issue that must be addressed [28]. A few studies indicated that experiments showed that network and user features were not sufficient for detecting rumors. The majority of relevant studies either relied entirely on content features or content-based features were among the features used to detect fake news along with other features [1,29].

#### 3.2.1 Content-based features

For news validation, news content (linguistics and visual data) is used as a feature in fake news detection models [30]. The results of research conducted by Kim, Kim [28] demonstrated that the accuracy of detecting rumors using only the content-based feature was higher than using all other features simultaneously combined. Human psychology favors articles with appealing multimedia materials coupled with text since readers believe them. When a news article contains multimedia elements, such as images instead of solely text, the news spreads quickly and reaches a large number of users [19]. Images and other visual content in text spread more quickly and receive 18% more clicks, 89% more likes, and 150% more re-tweets [31]. Therefore, even while text-based content is essential for verifying news, visual content has a vital function in fake news detection [18].

I Textual-based features

Textual content-based fake news detection studies are mainly dependent on the features extracted from the text that the classifier relies on in identifying fake news, such as linguistic features and syntactic features [32], sentiment features [33], or features based on the style and quality of the writing [5]. Experiments have indicated that fake news often carries strong or extreme sentiments to excite and attract the reader [5]. Moreover, fake news tends to be aggressive in expression, containing words with biased and violent emotions [34]. Fake news titles often include more exclamation marks and question marks than real news [35]. In addition, such news headlines often appear in capitalized words, or letters within a word are unusually repeated [36]. It also often contains repeated vocabulary, unlike real news, which consists of a variety of vocabulary. In addition, most fake news headlines are longer than real news headlines [37]. In contrast, the fake news article has a shorter content text than the real news article [38].

II Visual-based features

With the development and increasing use of multimedia, text news posted on social media platforms now contains visual content such as richly semantic images and videos. Text-based feature models struggle to deal with visual information due to the discrepancy between textual information and visual information [21]. Although visual features can be extracted from visual components, only a small number of research items have used visual features to identify fake news [30]. The text content and propagation structures are the main features used in traditional fake news detection methods. Posts on social media platforms contain rich visual data such as images and videos. Unfortunately, multimodal data are ignored by most researchers. Users find images more engaging than text articles since the visual material clearly conveys the events that interest them. To identify fake news, multimedia content, such as images and videos, is crucial. Visual diversity score, clustering score, similarity distribution histogram, clarity score, and coherence score are some of the most significant visual features of a given piece of visual news material [19]. Major developments in image processing have shown that images can simply be manipulated and transformed, making it easier to fabricate fake images. Consequently, investigating interconnections between multimodal data and creating models based on a fusion between these multimodal data may be an effective method for detecting fake news. Three types of fraudulent images related to disinformation on social media are prevalent: image mixing, image mismatching, and image manipulation [24].

• Image mixing occurs when images from past events are used to illustrate current events.
• Image mismatch happens when the text does not match the original image.
• Image manipulation or tampering includes maliciously modifying existing images.

For instance, Jin, Cao [39] discovered that real and fake news differs in visual information's statistical properties. In particular, a false event frequently includes a small number of images circulated regularly (limited by the source of the images), whereas the actual images associated with real events demonstrate high diversity. As a result, they suggest several visual and statistical features, such as the visual diversity score and the visual coherence score. These features are intended to describe variations in fake news detection. Salloum, Ren [40] also proposed the Multi-task Fully Convolutional Network (MFCN) model. The surface and border of samples used by this model extracted tampering features that could be used to detect local removal and splicing in images. Huh, Liu [41] used exchangeable image file format (EXIF) metadata to present a Convolutional Neural Network (CNN)-based fake images detection model, which assesses whether images can be produced by the same imaging pipeline or not. On the other hand, Wang, Tahmasbi [42] examined fauxtography images in social media postings and discovered that doctored images enhance user interaction on Twitter and Reddit. Their research showed that doctored images have a "clickbait" effect that increases engagement and are frequently used as memes to spread misinformation or satire.

#### 3.2.2 Social context-based features

**Fig. 3:**
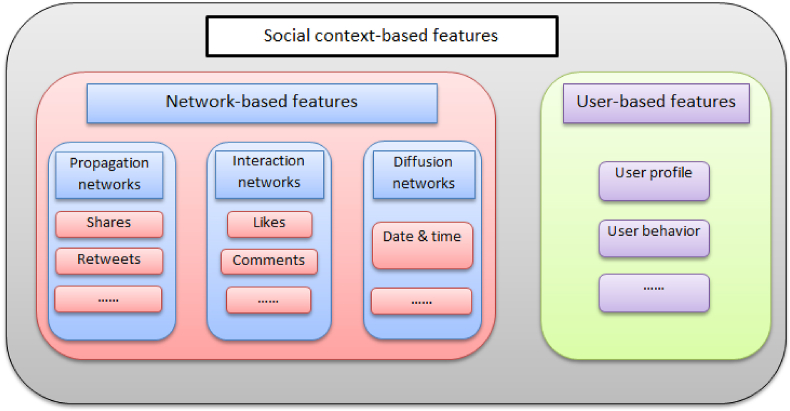
The social context-based features used to detect fake news.

The existing context-based social features used in fake news detection models are categorized into two types: Network-based features and User-based features, as shown in Fig. 3.

I Network-based features
  Network-based features are extracted by building specialized networks, such as propagation networks, interaction networks, and diffusion networks. Propagation networks-based features refer to a method of analyzing information spread and diffusion patterns via social networks to identify fake news [30]. Interaction networks-based features involve a method that relies on analyzing patterns of interactions between users and content posted on social networks to detect fake news [43]. Lastly, Diffusion networks-based features analyze how information spreads through social networks over time to detect fake news [44]. Some studies depend on network-based features, especially those related to rumor detection [1]. There are several network-based features used to detect fake news, such as re-sharing of the news [45], message pattern dissemination via social networks [46], temporal and spatial information about the spread of the message [47], and follow-follower relations [48]. Furthermore, the number of comments and likes that users respond to are also indicators of news credibility from the point of view of their interaction [1].
II User-based features

Features based on user characteristics can be used to detect fake news [32,48]. Social media users play a key role in spreading false news [49]. Therefore, the scanning and analyzing of users' profiles who post and share news can be leveraged to detect fake news [37]. User-based features can be categorized into the following characteristics.

• User credibility characteristics: These include the user's trustworthiness grade, account influence, and reputation, as well as the user's total amount of individual messages, friend-to-follower ratio, and number of followers and friends [50].
• User behavior characteristics: The aim of user behavior characteristics is to collect user behavior patterns, including genuine and fake users. User irregular values are characteristic of user behavior. In study articles for the detection of anomalous social network data, this is calculated by dividing the user's connections throughout a specific time period by the average utilization of the conversation mean [51].
• User profile characteristics: This indicates the details of the user profile, such as account name, location data, and registration data to assess whether the user account verified [2]. Other details include personal description, personal photo, and other personal information belonging to the user's account [1].

### 3.3 Modality-based approaches

Existing fake news identification research is classified into two groups: unimodal studies and multimodal studies. Extracting comparable aspects, such as textual and visual data, is a fundamental task in Natural Language Processing (NLP) and Computer Vision (CV). Moreover, it is also a crucial stage in fake news detection. In this section, we examine studies of unimodal and multimodal fake news detection.

#### 3.3.1 Single modality-based fake news detection

In order to identify fake news, single-modality-based approaches use a single type of data, such as text news content, visual content, and social contexts-based information. Unfortunately, single-modal data is insufficient for identifying fake news; thus, this group's techniques perform poorly. News textual content plays a significant role in establishing its veracity. Yu, Liu [52] suggested a CNN-based model to extract local-global relevant characteristics from text content. The two techniques focus on identifying false news at the event level, necessitating event labels, which raises detection costs. Ma, Gao [53] presented a generative adversarial network (GAN)-based model to learn indicative representations from enlarged text data to detect rumors. Qian, Gong [54] suggested a model that produced user feedback based on the article. This user response was then incorporated into the classification task together with information, both at the word level and at the sentence level, from real statements to solve the problem of lacking user feedback as an auxiliary source of data in the early detection of fake news. According to Giachanou, Rosso [55], emotional signals are utilized to distinguish between real and fake news. The authors suggested a Long-Short-Term Memory Networks (LSTM) model that combines emotional signals gleaned from the text of the claims. However, Cheng, Nazarian [56] proposed a rumor detection model using an LSTM-based variational autoencoder to extract latent representations of text at the level of tweets. In addition to text information, images are essential in fake news detection since they greatly impact the way news is spread [39]. The research presented by Raza, Munir [57] focuses on image-based counterfeit detection models. A method for detecting deep fake content called a deepfake predictor (DFP) was proposed in this study and was built on the VGG-16 and CNN architectures. This model was trained using the deepfake dataset, which included both real and fake faces. Several transfer learning methods were used, including Xception, NAS-Net, Mobile Net, and VGG-16. To identify fake news based on images, Qi, Cao [58] presented a Multi-domain Visual Neural Network (MVNN) framework, which fuses visual data from the frequency and pixel domains. Moreover, they used a multi-branch CNN-RNN model to extract visual features from several semantic levels in the pixel domain. Specifically, they created a CNN-based network to automatically capture complex patterns of fake-news images in the frequency domain. The representations of frequency and pixel features were fused by the attention method. Social context features were also employed for fake news identification on social media in addition to textual and visual features. In this context, Liu and Wu [59] proposed a method of extracting user attributes from user profiles to assess the veracity of the news as well as early detection of fake news.

#### 3.3.2 Multiple modality-based fake news detection

**Fig. 4:**
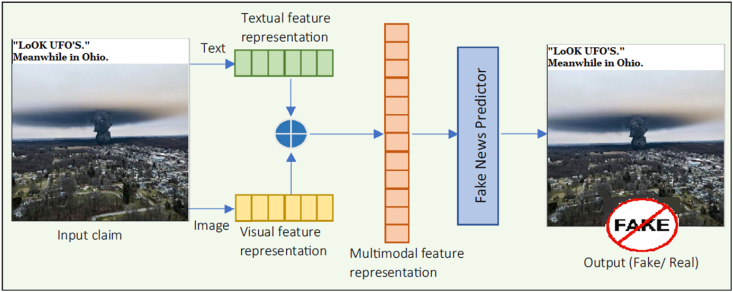
A general framework for multimodal fake news identification.

Visual and textual data are combined in multimodal approaches to identify fake news. In tasks involving fake news detection, both textual and visual indicators are effective. Currently, news on social networks frequently includes both written and visual content. Hence, it makes sense to combine them for improved performance of detection models [21]. Understanding how to effectively analyze data from many sources is crucial for multi-modal fake news identification, and several models rely on feature fusion and augmentation to boost performance. In order to learn further about shared multi-modal representations used in fake news detection, Khattar, Goud [60] converted the broad complementation framework into a multimodal variational autoencoder. They jointly learned the encoder, decoder, and fake news detector to train their model. By using Twitter and Weibo datasets in tests, they observed the detection accuracy to be 74.5% and 82.4%, respectively. Li and Lei [13] stated that creating efficient inter-modal interaction modules, such as the attention mechanism to identify fake news, is the core idea. In this regard, in order to create a joint representation, Jin, Cao [61] utilized social context, images, and text content. In this study, the visual representation was enhanced using an attention mechanism. Nevertheless, since the attention values were solely derived from the social-textual representation, the improved visual representation cannot reflect the similarity between the visual representation and the social-textual representation. Their experiments provided a detection accuracy of 78.8% on the Weibo dataset and 68.2% on the Twitter dataset. Zhang, Fang [62] utilized multi-channel CNNs with attention mechanisms to combine information from several sources. The authors concentrated on multi-modal data augmentation by emphasizing crucial visual regions while providing textual instruction. In this research, the accuracy was 86.6% on the Twitter dataset and 81.6% on the PHEME dataset. The hierarchical Multi-modal Contextual Attention Network (HMCAN) architecture was also presented by Qian, Wang [63] for joint modeling of multi-modal context information and hierarchical semantics of text in a deep and unified framework for fake news detection. The multi-modal contextual attention network models the multi-modal context of each piece of news, and the accuracy of their tests on the datasets (i.e., Weibo, Twitter, and PHEME) was 88.5%, 89.7%, and 88.1%, respectively. In contrast, Singhal, Shah [64] developed a multimodal model and employed pre-trained models, which are Bidirectional Encoder Representations from Transformers (BERT) in the first study, and XLnet in the second study, to encode text features before combining them with visual features extracted using the VGG-19 model. Despite the accomplishments of these pieces of research, none of them considered the intricate cross-modal connections contained by fake news, which limits multi-modal content identification efficacy. In their 2019 study, the researchers' findings were 77.7% for the Twitter dataset and 89.23% for the Weibo dataset. However, the detection accuracy of their model in their 2020 study was 85.6% for the Gossipcop dataset and 84.6% for the Politifact dataset. To identify fake news, some studies employed certain techniques to compare textual and visual features based on similarity. Similarity Aware Fake News Detection model (SAFE) was created by Zhou, Wu [65], which considers the similarity between the input image and text. This precaution is necessary since fake newsmakers may entice readers by including offensive imagery. Three primary elements compose SAFE's model: a cross-modal similarity extractor, a fake news prediction module, and a multimodal feature extractor. By working across multiple modes, multimodal feature extraction captures both textual and visual content. The cross-modal similarity extraction module employs a cosine similarity metric to infer the connection between news text and news images. Furthermore, a softmax layer is used in the fake news prediction module's final prediction. Based on the text, images, and degree of misalignment, this method determines whether a given piece of news information is real or fake. Two standard datasets from PolitiFact and Gossip Cop were used for the tests. For both datasets, the accuracy based on the F1-score measure ranged from 75% to 85%. The basic framework of the multimodal fake news detection model is illustrated in Fig. 4.

## 4 Critical analysis of fake news detection studies

To highlight the challenges and limitations of fake news detection models and identify their weaknesses, Table 2 shows the relevant studies in English that provided models (unimodal and multimodal) for detecting fake news for the period 2018–2023 based on the following criteria.

• The results were weak or could be improved.
• The study had one or more of the following topics: Dataset, overfitting/underfitting, image-based features, feature vector representation, machine learning models, and data fusion.

**Table 2:** Critical analysis of fake news detection studies.

Authors & Year	Dataset	Features	Methodology	Result	Limitation
Ghanem, Rosso [66]	They used a highly imbalanced FNC-1 dataset of fine-grained labels.	Titles and articles	They presented an NN-based detection model and used a combination of N-grams, Google News word2vec, and lexical cue words for feature representation.	59.6% F1-score	The issue of the imbalanced dataset, and its strong bias towards the majority classes over the rest (misclassification), was not addressed. In addition, simple techniques were used to extract features.
Elhadad, Li [67]	LIAR, ISOT, and FA-KES datasets were used for experiments.	News title, content, publisher and other metadata	Feature selection techniques were used before Term Frequency- Inverse Document Frequency (TF-IDF) and N-grams were used for feature representation. Several Machine Learning (ML) algorithms, such as Support Vector Machine (SVM), Decision Tree (DT), Logistic Regression (LR), etc., and NN, were employed to classify fake news.	Accuracy of 62% on the LIAR dataset, 96% on the ISOT dataset, and 58% on the FA-KES dataset	They did not use DL techniques. In addition, using a small dataset like FA-KES may lead to overfitting/underfitting. Furthermore, the use of feature extraction techniques, such as TF-IDF, and N-Gram, leads to the loss of many features and neglects to capture semantic relationships between words.
Kumar, Asthana [36]	They used a dataset of 1356 news instances consisting of 300 tweets from Twitter. In addition, they used instances of the FakeNewsNet dataset of 1056 examples. The FakeNewsNet dataset was retrieved from PolitiFact.com.	News title (text)	They proposed several ML and DL-based detection models to identify fake news. A pre-trained Glove for word vector representation was also utilized.	Results ranged from 57.58 to 58.68% for DL models and 73.29–88.78% for ML models. The most promising result was CNN + A Bidirectional LSTM (Bi-LSTM) with an attention mechanism.	There was a variation in the results even within the same models of ML or DL, where the result of testing in most of the models on the test data was approximately 97%, but after these trained models were tested on a larger PolitiFact dataset, the results were poor. Note that the PolitiFact dataset has the same features as the one used to train the models. This means that their proposed models fall into the problem of overfitting, which prevents them from generalizing new data. It should be noted that the FakeNewsNet dataset is multimodal and visual features were excluded from their research.
Shu, Mahudeswaran [68]	FakeNewsNet was used, which is a multi-modal repository that consists of two combined datasets: PolitiFact (1056 examples), and the imbalanced GossipCop (22140 examples).	News text and social context	They conducted several experiments using several ML-based models, such as SVM, LR, and Naive Bayes (NB), and two DL-based models CNN and LSTM, to explore some of the features of the proposed dataset to detect fake news. A one-hot encoded vector for word representation was used.	The accuracy ranged between 58 and 9.1% on the PolitiFact dataset and between 49.7 and 69.8% on the GossipCop dataset.	Since the features were not well represented in detecting fake news, which was represented as a one-hot encoded vector, the results of experiments were poor. In addition, one of the merged datasets (PolitiFact) was small in size although rich in features, which may lead to overfitting that reduces the generalization of the model when tested on test data.
Raza, Ding [69]	The textual content of two datasets has been merged. They used the NELA-GT-2019, a large-scale, multi-labeled dataset. In addition, Fakeddit was also utilized, which is a large-scale, multi-modal, multi-labeled dataset.	News content, social contexts, and comments	They presented a model based on the transformer architecture. Based on past observations, the encoder learns useful representations of fake news, and the decoder predicts future behavior. BART was used as a sequence-to-sequence transformer with a bi-directional encoder and an autoregressive left-to-right decoder. Multi-headed attention was also used to assess the significance of different segments of text.	74.8% accuracy	Two multimedia datasets were used; however, visual features were neglected. Moreover, the issue of an imbalanced dataset was tackled through the under-sampling method, as the omitted examples may have contained relevant information regarding the predictive class. In addition, a pre-trained word embedding model was not used to enrich the features.
Gôlo, de Souza [70]	They used a fake news class combined from six datasets: four in English and two in Portuguese	Text	They proposed a multimodal representation method based on variable autoencoders to combine extracted semantic, linguistic, and topic features to represent fake news. They used different models to extract textual features. Next, they explored various ways of fusing them and making a decision.	The results for different models on the six datasets were as follows: 40.7%, 65.6%, 70.1%, 70.7%, 86.6%, and 95.2%, based on the Fi-score.	Although the lack of significant and balanced standard datasets prompted researchers to use the One-Class Learning method, it has several disadvantages. It is not suitable for a critical domain where accuracy must be considered. This method is disadvantageous as it is applied to binary classification. In addition, this single class requires feature-rich examples that cover all instances of this class. Since the opposite class is not represented, it leads to misclassifications and poor generalization of the unseen data (i.e., the problem of OOV). Moreover, some datasets contained visual features and have been neglected.
Choudhury, Acharjee [71]	They used two datasets: the LIAR dataset of 12,836 examples, and the imbalanced Fake Job Posting dataset of 18,000 job descriptions	Text	They used TF-IDF for text vectorization and examined several ML classifiers, including NB, SVM, LR, and RF. They also utilized GA to find the best solution.	The accuracy was 60% on the LIAR dataset and 97% on the Fake Job Posting dataset	The results depicted a discrepancy between the different proposed ML models on the two datasets. This was particularly evident in the LIAR dataset as well as the two datasets of Fake Job Posting. While the LIAR dataset is fine-grained, binary classification is adopted through the proposed methodology. The reason for the high scores in the Fake Job Posting dataset is that they used only 800 examples of fake news out of 20,000, indicating a severely imbalanced dataset. The test set was not represented in a balanced way. In addition, the ML-based models proposed by them, and also the use of TF_IDF for vector representation, were not comparable to the effective performance of DL models and pre-trained models for feature extraction.
Wang, Ma [72]	They conducted experiments on two datasets, Twitter and Weibo. Weibo consists of 19,056 news stories with attached images. The imbalanced Twitter dataset included 13,924 text tweets with 514 images.	Textual and visual features	They proposed an EANN model consisting of three major modules: the multimodal feature extractor, the fake news detector, and the event discriminator. They used CNN for extracting textual and visual features. Pre-trained VGG-19 was also used for visual feature representation. For fake news identification, Softmax was implemented.	The accuracy value was 71.5% on the Twitter dataset and 82.7% on the Weibo dataset.	In their research, it was indicated that any tweet that does not contain images, duplicate tweets, or tweets that contain images that are not of high quality had been deleted, which implies that the Twitter dataset used was less than 514 examples. This is insufficient for a model based on Deep Learning (DL) techniques. In addition, the researchers did not use a pre-trained word embedding model for text-based feature extraction. Furthermore, they directly combined textual and visual features.
Khattar, Goud [60]	They used two multi-modal datasets: the Twitter dataset consisting of 17000 tweets, and the Weibo dataset consisting of 6152 news articles with text and images. Both datasets were in Chinese.	Text and images	Word2Vec for word representation and VGG-19 for image representation were used. The textual encoder consists of LSTMs, and the visual encoder was composed of two fully connected layers. The decoder was made up of the same dimensions as the encoder layers.	74.5% accuracy on Twitter and 82.4% accuracy on Weibo.	Translating the dataset from one language to another may lose news characteristics, especially since fake news contains unique features. These features include certain vocabulary that the other language may not accurately cover in translation, in addition to writing style, etc.
Singhal, Shah [64]	They used Twitter and Weibo datasets	Text and images	They used BERT to extract text features and VGG-19 to extract visual features. In a multimodal fusion phase, they fused representations collected from various modalities.	An accuracy of 77.77% on Twitter and 89.23% on Weibo.	The extracted features were fused based on simple concatenation.
Giachanou, Zhang [73]	They used three datasets: MediaEval, GossipCop, and a small sample of the PolitiFact dataset of 2779 examples.	Textual, visual features, and sentiment polarity of text	They produced a model based on NN that combined textual, visual, and semantic information. The textual features are extracted by the GoogleNews-vectors-negative300 model, and a Valence Aware Dictionary and sEntiment Reasoner (VADER) lexicon were employed to compute the sentiment score. To extract image tags, they used pre-trained models VGG16, VGG19, Residual Network (ResNet), Inception, and Xception. Cosine similarity was applied to calculate text-image similarity.	The F-1 measure was 62.2% on the MediaEval dataset, 82.9% on the GossipCop dataset, and 92.5% on the PolitiFact dataset.	The pre-trained GoogleNews-vectors-negative300 model was unable to address OOV words. In addition, one of the datasets used (PolitiFact) was very small. Moreover, the issue of imbalanced classes for the GossipCop dataset was resolved by using under-sampling, which is an inefficient method that may result in the removal of significant features. It is also clear that the model's overfitting problem was overcome by setting high values for the dropout layers.
Song, Ning [74]	They used four multimodal datasets: A small sample from Twitter, Weibo A, Weibo B, and Weibo C.	Text and images	They used Wor2Vec for word embedding and VGG-19 for image embedding. A CARN layer reinforced the representation of the target modality's features by arbitrarily extracting data from the source modality. A CNN-based MCN layer reduced the impact of noisy information that the CARN layer may produce and extracted the final textual feature representation. A Softmax-based fake news prediction layer was also used.	In terms of accuracy, Twitter scored 74.1%, Weibo A scored 85.3%, Weibo B scored 86.9%, and Weibo C scored 92.2%.	The Twitter dataset consisted of only 514 tweets containing an image. We noted a discrepancy in the results according to the dataset used despite the use of the same proposed model. This indicates the importance of the dataset in terms of size, quality, and diversity.
Jing, Yao [75]	They used the Gossip and Weibo datasets. Note that the Gossip dataset did not include images.	Textual (Tweet + reply) and visual features	To detect fake news efficiently, they developed a transformer-based method that learns the co-embedding of multimodal data (text, visual, and commentary). They utilized the Faster-RCNN model for visual feature extraction and the BERT model for text-based feature extraction.	The accuracy was 85.5% on the Weibo dataset and 85% on the Gossip dataset.	It is not a good idea to combine tweet text features directly with comments. Sometimes, comments are not related to news and are considered noise. In addition, the maximum limit for a tweet is 30 tokens, and when it is combined with a maximum of 10 comments; the maximum limit for each comment is 30 tokens. This leads to tweet features fading among comments features. In addition, the Gossip dataset does not contain images, although their proposed model addresses multimodal data.
Wu, Zhan [76]	They used the Twitter dataset (2016) and the Weibo dataset (2017).	Text and images	They extracted spatial-domain features by VGG-19 and frequency-domain features using CNN. Additionally, textual features were extracted using BERT. Hence, based on a fusion strategy with several co-attention layers, the visual features were combined before the textual features.	The accuracy was 80.9% for the Twitter dataset and 89.9% for the Weibo dataset.	They used two small datasets. In addition, one is imbalanced, and the other contains short tweets.
Segura-Bedmar and Alonso-Bartolome [77]	A fine-grained multimodal Fakeddit dataset was used.	Text and images	They used the multimodal model based on CNN architecture.	An accuracy of 87% was observed.	They did not use a pre-trained model to extract visual features. In addition, the extracted features were simply concatenated. Moreover, they indicated that their model suffered from misclassification because of class imbalance.
Wang, Mao [78]	They used a multimodal Weibo dataset of 6152 tweets.	Text and images	They used a RoBERTa as the text feature extractor and the CNN with a pre-trained VGG-19 as a visual feature extractor. Textual and visual features were combined in a fine-grained manner using scaled dot-product attention at the feature fusion stage.	An accuracy of 88.5%.	They only used tweets with images attached; hence, the dataset used was small.
Hua, Cui [79]	A ReCOvery multi-modal dataset containing 1859 COVID-19 examples was used.	Text and images	They presented a framework consisting of 1) data expansion related to the text based on back-translated, 2) multimodal information encoding based on pre-trained BERT for text encoding and the ResNet model for image encoding, 3) feature fusion, and 4) joint learning based on multi-headed transformers.	An accuracy of 80.5% based on Macro F1.	They used a small and imbalanced dataset consisting of 1297 examples of real news and 562 examples of fake news. They indicated that only topics related to titles and abstracts were retrieved to reduce the noise generated. Unlabeled positive examples may also lead to bias in terms of real news. This is because real news published online is often more frequent than fake news. Moreover, only textural features have been enlarged to enhance feature richness.
Kalra, Kumar [80]	They used an imbalanced Fakeddit dataset of six-way labels.	Text and images	For text and image feature extraction, they employed DistilBERT and VGG16 models, respectively. For the multimodal fake news detection task, they combined these extracted features by concatenation and then added a stack of dense layers.	An accuracy of 60.38%.	They indicated that their model performed poorly. Their model was overfitted, and a dropout layer was added after each layer due to the dataset used containing imbalanced classes. Further, fusing multimodal features from different models directly without taking advantage of each model's features and common features leads to a loss of several characteristics.
Jing, Wu [81]	They used two datasets. Weibo in English consisted of 7021 fake news and 5974 real news, while Twitter in Chinese contained 9528 examples.	Text and images	They used BERT for extracting textual features, and SwinT to extract spatial semantic features from images. They adopted progressive fusion based on Mlp Mixer as a fusion module for images to fuse feature information between modalities at a finer granularity. The Softmax layer was then employed to classify news.	The accuracy was 83.8% on the Weibo dataset and 83.3% on the Twitter dataset.	Despite the attempts to explore the most efficient methods to extract visual features and fuse them with textual features through several stages, the increase in results was minute. The results of one of the baseline studies compared were higher than the results of their proposed model. The study used one small and one imbalanced dataset, dating back to 2014 to 2016. Dropout layers with a value of 0.5 were used to prevent overfitting.
Wang, Feng [82]	They used multimodal Fakeddit and Weibo datasets.	Text and images	They proposed a multi-modal transformer using two-level visual features for fake news detection. To extract image features, they utilized ResNet and for text embedding, they employed Faster RCNN. They also used N-layer transformer encoders to acquire a high-quality representation and classify the news based on a fully connected layer.	The accuracy was 87.66% on the Weibo dataset and the accuracy on the Fakeddit dataset was 91.88%, 91.62%, and 89.82% based on two-way, three-way, and six-way classification, respectively.	To address the imbalance of the Fakeddit dataset, they tested two methods, one of which was class-balanced sampling while the other was the re-weighted loss function, both with disadvantages including information loss, overfitting, and limited generalization. This was evidenced by the variation in the results, especially in the six-way classification.

**Fig. 5:**
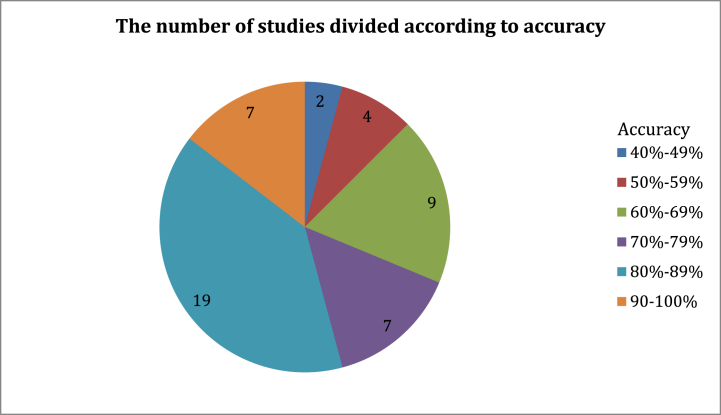
The number of studies divided according to the accuracy of their results.

**Fig. 6:**
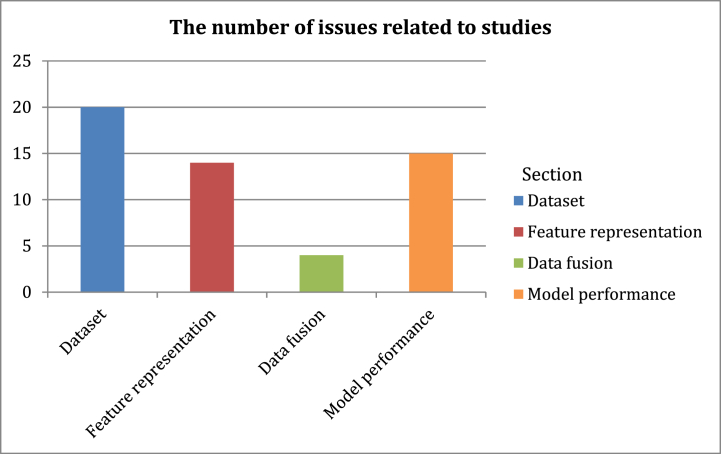
The main sections responsible for the problems generated and the poor results.

**Fig. 7:**
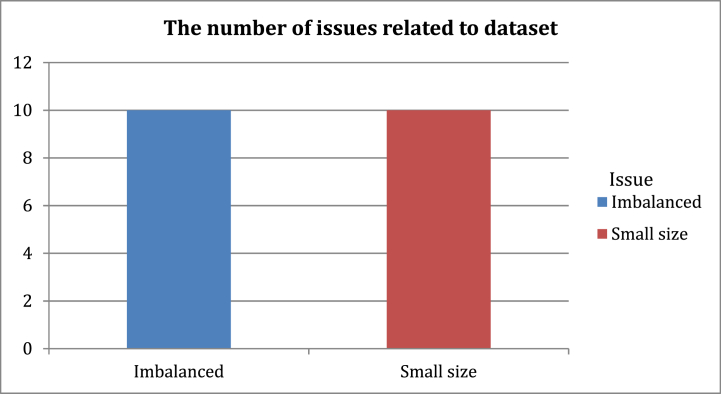
The number of issues of the dataset responsible for the problems and poor results in previous studies.

**Fig. 8:**
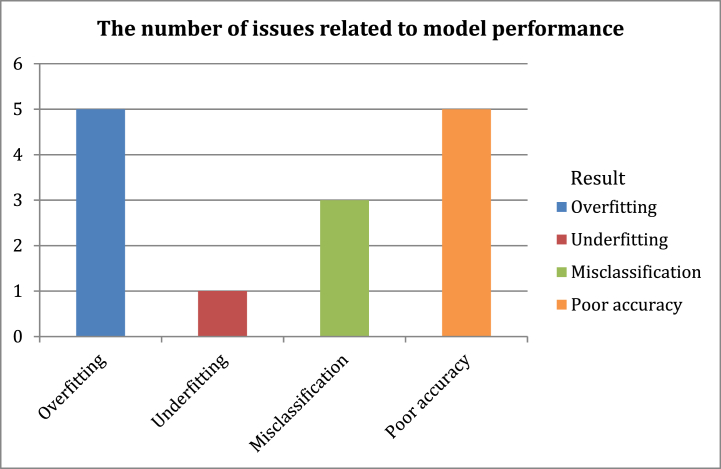
The results generated by fake news detection models.

The following figures represent statistics resulting from the analysis of the relevant studies in Table 2. Fig. 5 illustrates the number of studies divided according to the accuracy of their proposed models' results. We note that several studies require improvements for more accurate results. Fig. 6 shows the number of issues associated with the main sections of the fake news detection models in relevant studies. Fig. 7 illustrates the number of issues related to the dataset in relevant studies. These main sections of the fake news detection models in the studies analyzed in Table 2 led to undesirable results as depicted in Fig. 8. Most of the issues related to the model's performance as shown in Fig. 8**,** such as overfitting and misclassification, are related to problems with datasets. The use of machine learning models did not provide high results in this domain. In addition, the poor representation of features and not employing efficient pre-trained embedding models may not address the problem of Out-Of-Vocabulary (OOV) words Also, the exclusion of visual features from their use in detecting fake news in detection models has contributed to reducing the efficiency and accuracy of the classifier. Furthermore, directly fusing text and image features may lead to a loss of relationships and attributes that may increase detection accuracy. The number of problems and results may differ from the number of studies discussed in the previous table, as many studies conducted experiments on more than one dataset, and each dataset depicted certain results. In Section 5, the most significant limitations associated with fake news detection models will be discussed, as well as their consequences and the effective methods to address them.

The limitations of this review are that some studies showed high results even though they faced one or more of the problems addressed in this review, and these studies did not disclose the way in which this problem was addressed.

## 5 Challenges of fake news detection models

Based on the relevant studies and their results, and despite presenting several models based on AI approaches to detect fake news on social media platforms using various effective methods and features, the results still require improvement to enhance accuracy. The following challenges and limitations affect the performance of these models as well as the accuracy of fake news detection.

### 5.1 Datasets used

**Fig. 9:**
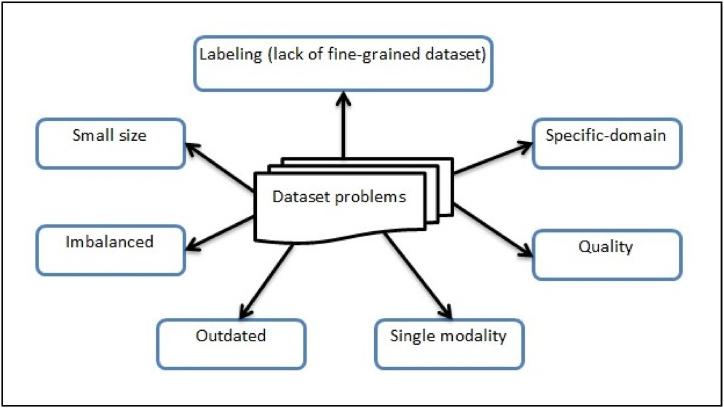
Problems associated with fake news datasets.

Since the supervised models for detecting fake news are being examined in this study, the most significant part of training these models and increasing their efficiency in detecting fake news is the dataset. When detecting fake news, datasets are just as crucial as techniques. Since a lack of data is the primary issue in identifying fake news, more data should be made available on identifying fake news. Model performance increases with training data [30], and strong positive correlations exist between the sample size and the technique's accuracy in detecting fake news. This emphasizes the significance of testing fake news detection models using several samples. Moreover, a significant factor in variability was the sample size used to define the detection accuracy [83]. Several datasets are available in the field of fake news detection; however, there are some restrictions, such as size, modality, and granularity, in addition to being outdated [69]. Most of these standard datasets are small and binary (real and fake) labeled. As is the case in the health domain, especially in the detection of misinformation regarding Covid-19, the two studies by Alghamdi, Lin [84] and Wani, ELAffendi [85] indicated that the most serious challenge they faced was the small size of the datasets related to Covid-19. However, fake news has many forms. Fakeness varies in severity. In reality, a news article may contain both true and incorrect statements. Therefore, it is crucial to categorize fake news into different groups according to disinformation degrees [20]. These issues severely hamper research and fake news identification. Thus, one may frame the issue as a multi-class classification task or even a regression task rather than a binary classification. Likewise, fake news would be given a higher priority for preventative measures [16]. Another difficulty is the data source, as unstructured data contains extraneous information and incoherent values, which might degrade algorithm performance. Although some datasets contain data from multiple domains, many contain data from specific domains such as politics [68], society [86], technology [87], health [88], and celebrity gossip [89]. Due to the small number of categories, these data samples can include limited contexts and writing styles. Most fake news datasets include text information; thus, the majority of relevant studies focus on employing text data in detecting fake news. These text datasets include LIAR [90], BuzzFeed News [91], Fake News Challenges (FNC-1 dataset) [92], and Some-Like-It-Hoax [89]. There is a dearth of multi-modal datasets in this problem space [20]. Today, people consume information online through a variety of multimedia sources, including images and videos, which serve as supplements to the text. However, images and videos can also take the form of fake news. Benchmark datasets, like ImageNet for visual object identification, are still lacking in fake news detection. The size and variety of fake news image datasets are limited, necessitating the need to study them further. Image characteristics provide models with additional data that can greatly aid in the detection of fabricated images and image-based fake news [93]. Multimodal datasets are really needed, which combine several modalities like text and image. One of the most pressing open problems is the development of multimedia datasets for fake news detection. Not many attempts have been made to include non-text news, such as images and videos. Therefore, more work should be done to broaden the modality set while creating fake news datasets [17]. Additionally, given that different data sources exhibit different styles and concentrate on various issues, we must also pay attention to datasets gathered from various platforms, such as Reddit and Instagram. For instance, Reddit encourages the free exchange of information, which might make content filtering harder [16]. Unfortunately, the collection of fake news data requires a lot of time and effort, which leads to poor standards [24]. A few attempts have been made to address the smallness of the dataset in previous studies by merging two datasets and forming a larger and more diverse dataset [68,69]; however, the variation may be wide between these two datasets, in addition to the different structures of these two sets, which complicates the merger process. One of the successful methods of increasing the size of the dataset, which is widely used in image processing, is the data augmentation technique [94,95]. Furthermore, since it is important to detect fake news early to prevent spreading, analyzing and addressing this issue in a weakly supervised learning approach—that is, with few or no training labels—becomes crucial because gathering the ground truth and labeling a dataset requires plenty of time and effort [20]. Fig. 9 depicts the main problems with fake news datasets.

### 5.2 Overfitting and underfitting issues

Machine Learning (ML) and Deep Learning (DL)-based models face significant challenges from overfitting and underfitting. It is true that this problem is not limited to a specific field and involves various tasks. However, based on previous studies that presented fake news detection models, especially those that use deep learning techniques, as well as the lack of large and balanced datasets about this task, these models suffer from overfitting and underfitting, which impact the model's performance [96]. When ML or DL models are overfitted, they learn in a way that is not applicable to the training samples and are no longer generalizable to the entire population [97]. Some cases that lead to overfitting include 1) insufficient training data, which lacks enough examples to adequately cover all possible input data values [98], and 2) large volumes of insignificant information or noise present in the training data [99]. In addition, if the model was trained with an imbalanced dataset, it may be overfitted to the training examples from the underrepresented classes and might not generalize well on the test set [100]. With respect to the underfitting issue, when the model cannot successfully represent the links between input variables and output variables, it leads to underfitting, resulting in an increased error rate in both the training set and the test set (the unobserved dataset) [101]. Since under-fitted models are the result of biased data, they produce incorrect results for both the training and the test data. On the other hand, high variance data results in overfitted models, which produce accurate results for the training set but not for the test set [102]. Therefore, training data should ideally reflect the entire population. Most of the models based on deep learning, especially models that target fake news detection, employ several methods to prevent the overfitting issue, such as reducing model complexity by using fewer neurons or layers and implementing regularization techniques (L1, L2), weight decay, or batch normalization and dropout [73,80], which limits the performance of the model and thus affects the accuracy of fake news detection. Regarding models of fake news detection that suffer from overfitting due to the use of an imbalanced dataset, the issue of overfitting was addressed in a few previous studies by the under-sampling method [69,73]. Nonetheless, this is a traditional and ineffective method, since there may be examples containing relevant features that will be removed. In order to effectively use the limited training data of the available datasets and avoid overfitting, researchers have created inventive workarounds. Generating image variants from the original dataset through a technique called data augmentation can help in artificially increasing the size of a training dataset [99]. Data augmentation techniques can also be used to mitigate class imbalance that leads to overfitting by increasing the number of examples of the few classes [100]. Recently, data augmentation methods have also been used to generate text examples [103]. More training data collection is the most efficient strategy to reduce overfitting [97].

### 5.3 Image-based features

The two main data sources for fake news detection are news content and social context. With respect to content, visual-based and linguistic-based approaches are used to extract features from data material. In many natural language processing tasks, such as text classification and clustering, linguistic features have received extensive research. In addition, they are used for specialized applications, such as deception detection and author identification. However, the usage of image-based features has not been investigated often in the literature on fake news detection [104], despite the image carrying abundant semantic information [105]. Additionally, it is demonstrated that visual features derived from images include significant markers of fake news. Few studies have examined the use of efficient visual features in the fake news identification issue, such as conventional local and global features [106]. However, as more people have access to image editing tools, visual features that can differentiate between real and fraudulent content are becoming more crucial and might be investigated in additional detail [104]. The use of "manipulated" images in fake news posts was more prevalent in terms of containing artificial text, and the potential for these images to contain memes, text, or face alteration. The researchers discovered specific commonalities in the images that fake news posts frequently employ, even though the images themselves may not have been altered. For example, darker hues evoke more negative feelings than lighter hues. This suggests that false news articles purposely employ images that make their readers feel anger, fear, or suspicion, further inciting an emotional response. Fake news is also more likely to have violent images with a higher probability of including blood and gore. According to studies, text and visual elements complement one another to influence readers' perceptions, knowledge, and beliefs about a particular subject. It cannot be denied that textual and visual analysis can provide insight into the detection of fake news. However, the way in which images and text are framed in relation to one another influences people's reactions. This provides a compelling argument for using images and linguistic features as complementing signals to identify fake news in the context of the fake news detection [107]. Most previous fake news detection studies used textual features. Therefore, in order to create a stronger and more reliable model, an investigation of image features should be conducted [30].

### 5.4 Feature vectors representation

**Fig. 10:**
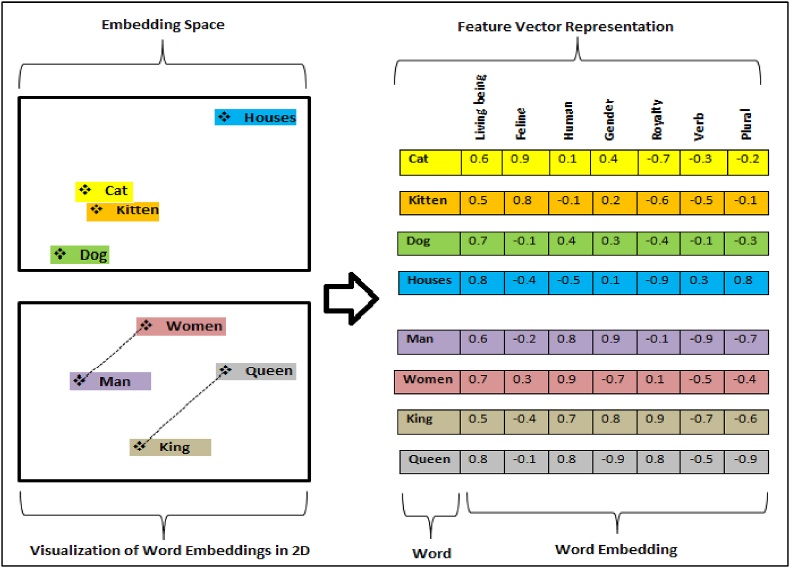
Feature vector representation.

While analyzing deep learning-based models provides a higher accuracy rate than other techniques, feature extraction and representation are crucial to improving their performance and accuracy. Choosing appropriate feature extraction techniques can also affect fake news detection accuracy. Research projects must consider the most appropriate classification method for particular features. For instance, processing long text features is essential for sequence-based models. Therefore, to enhance performance, much attention should be paid to the selection of classifiers and features [108]. News articles have transitioned from text to multimodal due to social media growth. Since news articles now include images, it is necessary to create a model that is based on a multi-modal dataset. Early studies have proposed several fake news detection models, most of which rely on textual features and are hence inappropriate for learning multimodal (text and image) shared representations [109]. Image processing may use gradient dimensions, RedGreenBlue (RGB), color histogram, color correlogram, color wavelets [110], color intensity, edges, regions, and other properties [111]. Feature vectors are particularly popular for image processing due to the simplicity with which properties of an image can be mathematically evaluated when located in vectors. While using image-based data for ML and DL models is simple (for example, using RGB values as input), text data is more complicated. The analysis of various features of fake news' multimodal nature has received little attention. This is despite multiple models being developed to address this issue, as working in a multimodal environment is challenging in itself. Although some of the suggested methods have been effective in identifying fake news, they still face challenges in assessing how diverse features from various modalities relate to one another [20]. Feature extraction techniques extract a collection of features, known as a feature vector. This maximizes the prediction rate with a fewer number of elements and produces a feature set similar to several instances of the same symbol [112]. Thus, there is a need for effective methods such as vectorization—also known as word embeddings in the NLP field—to transfer text input into numerical data. ML and DL learning models can also be built using these vectors. Moreover, two different methods can turn text input into numeric vectors: 1) frequency-based or statistical-based word embedding techniques, such as One Hot Encoding, Count Vectorizer, Bag-of-Words (BoW), N-grams, TF-IDF, etc., and 2) prediction-based word embedding models, such as Word2vec, GloVe, Fasttext, BERT, etc. In the first method (frequency-based or statistical-based word embedding techniques), it is a daunting task to turn text into numeric vectors when a huge corpus is present as the vector's dimension is directly dependent on the number of unique tokens. Using less space, it might eliminate some of the least frequent tokens. However, doing so would also remove some potentially relevant information. In any case, there can be different documents with the same feature vector, and there is no relationship between tokens in the bag of words. Hence, similar vectors are created. One of the common and effective techniques used in deep learning models to extract features for vector representation is the prediction-based word embedding model, wherein each word is converted into a vector represented semantically and grammatically via these word embedding models [113], which identifies relationships and similarities between words in the text corpus. In other terms, word embeddings pack more data into fewer dimensions (low-dimensional vectors include words with similar meanings) [114]. The distance among embedding vectors indicates the proximity of words to each other based on their relationships [115]. For example, as shown in Fig. 10, the words "cat" and "dog" are semantically related because they belong to the same class of the family of mammals. In addition, the words "woman" and "man" are placed close together.

When unknown words are not gathered to create the vocabulary in the training stage, or if they are disregarded owing to low frequency across the corpus and occurred in the test, the performance of most of the prediction models for many NLP tasks declines. These words are known as Out-Of-Vocabulary (OOV) words, and the inability of representation models to effectively train a representation for them might weaken NLP tasks performance, including models for detecting fake news. Some documents have noise (as in the datasets with social media posts) [116], or have several technical, medical, or domain-specific terms, such as in the Bio-NER dataset [117]. These documents abound with OOV words. Consequently, the prediction model's ability to comprehend this statement may be limited by the absence of representation for these words. Simple tactics may be used to handle OOV words in distributed representation models. For example, OOV words can be ignored during task creation, since no word embedding representation has been learned for them. Since the representation model is unaware of the absence of a word, this method causes the prediction model to fit data without being aware of it. In these situations, either a separate random vector for each OOV word or a single random vector for all OOV words might be used. Some word embedding models, such as Word2Vec and GloVe models, cannot comprehend out-of-vocabulary (OOV) words or words absent from training data; however, more sophisticated models, such as BERT, can handle this problem [118]. Nonetheless, embedding methods can extract textual features and have the potential to develop better representations. With respect to the image domain, relationships between visual concepts in images, as opposed to relationships between words in the text, are crucial for Computer Vision (CV) tasks; however, they are challenging in terms of capturing. For instance, the model intends to infer the intricate connections between the numerous objects in an image to produce a description. As a result, numerous works have developed various vision encoders to represent these relationships and object properties [119]. Before the advent of Deep Learning (DL) approaches, it was necessary to employ feature extraction methods to gather pertinent data from divided regions and create automatic classification models based on these features. These methods often rely on shape, texture, or color descriptors. Examples of commonly used techniques include statistical moments, Histogram Orient Gradients (HOG), Gabor-based descriptors, and Fourier-based descriptors. Additionally, dimensionality reduction was applied to data before classification using Principal Component Analysis (PCA) [120]. It has been demonstrated that the object detection network's output can be significantly influenced by the chosen feature extractor [121]. Some pre-trained models related to image processing also exist, such as AlexNet and VGG-16, which face challenges in feature extraction. The VGG16 network model's initial input size is 224 × 224. If the image resolution is too high, it will increase the computational load on the computer's memory while training the network model. However, if it is too low, the VGG16 model will not fully extract the features. When compared to other models, the AlexNet model has a very shallow depth, which makes it difficult to learn features from image sets [122]. Nonetheless, some pre-trained models provide high image recognition accuracy, such as VGG19, ResNet, and Inception. Finally, based on the importance of the representation of feature vectors and their impact on model performance, there are a number of previous studies in the field of fake news detection that do not consider the selection of effective methods for extracting features and accurately representing them. Since these detection models handle social media data, which may contain noise. Therefore, a poor representation of the feature vector leads to a decrease in detection accuracy.

### 5.5 Models based on machine learning

Many previous studies presented models for detecting fake news using Machine Learning (ML) techniques [32,123,124], and although these techniques have provided promising results in several areas [125], their use in detecting fake news may not provide the desired results, especially as this critical field requires efficient models that provide high detection accuracy. An essential area of artificial intelligence is machine learning. Although machine learning has evolved over the past 20 years, several issues remain to be resolved [126]. These issues include complicated image interpretation and identification, natural language translation, and recommendation systems. The traditional artificial method of obtaining image information has not satisfied the needs of applications in related domains since the arrival of the era of big data and artificial intelligence. The model's low classification performance is caused by standard machine learning's inability to analyze huge datasets. This is due to the difficulty in optimizing feature design, feature selection, and model training. Consequently, standard machine learning-based data classification techniques are impacted in a variety of application sectors [127]. Unfortunately, shallow standard machine learning approaches have limited representation ability, and are insufficiently capable of extracting high-level knowledge from social data. Furthermore, shallow models rely heavily on manually created features, which are less adaptable [13]. Due to the difficulty and long duration of feature extraction tasks, biased features may occur. Fake news detection has not shown notable results using ML techniques, and the curse of dimensionality is caused by ML methods' creation of high-dimensional representations of linguistic data [30]. The current machine learning's main drawback is the inability to get systems to operate effectively on a big scale (i.e., outside of small- and medium-sized retrospective datasets). It is imperative to note that this is not a simple task since the effectiveness of a particular machine-learning method is greatly dependent on the quality of the input information (features). These features are typically created manually by a human domain expert via trial and error since they rely heavily on in-depth topic knowledge. Since feature engineering becomes a crucial, time-consuming, and exhausting stage in the machine learning process, the feature dimensions are excessively large, and the model efficiency is poor [128]. The training datasets used to train machine learning models have a significant impact on classification accuracy, and deep learning offers superior feature learning capabilities than conventional machine learning algorithms based on statistics. In addition, the features learned have more significant data characterization abilities, which is helpful for visualization and classification [129]. Deep learning produces accurate features that classify text and images. It further uses neural network models to analyze large amounts of data and extract latent features [127]. Moreover, DL algorithms have been found to outperform ML algorithms [130,131], and have provided remarkable advances in image analysis and text classification, thanks to the ability of the deep neural network efficiently to extract features and learn successfully [4,23], in addition to having a high ability of capturing complex patterns [4,132].

### 5.6 Data fusion

Not many studies in fake news detection utilize feature fusion. Hence, integrating data from several modalities would be helpful in determining whether news items are fraudulent [30]. However, integrating data from several modalities to identify fake news is a challenge that presents issues. Dealing with data from multiple modalities while maintaining correlation poses an inherent challenge when handling multimedia data. Furthermore, the association between text and image in a multimodal social media post is crucial to detecting fake news. Instead of simply merging the features, the transformer encoder may automatically fuse several data modalities, which can produce deep fusion features for fake news detection by efficiently learning the cross-modalities link between text and image [75]. Another difficulty is to propose a method for methodically combining various modalities to ensure that one modality supports the others. The majority of current research uses fusion mechanisms, which may not result in complementarity between modalities [16]. Despite the significant progress made in prior studies in other fields, a crucial topic of how to retain the unique features of each modality during fusing pertinent data between different modalities in fake news detection models remains ignored There should be a distinct difference in how representations of text-based or image-based features are learned, and it is not wise to directly fuse the feature representations of the different modalities into a single vector. Separate representations of features from different modalities will fail in fusing correlated and complementary data between various modalities [74]. Unfortunately, due to their disregard for inter-modality interactions, the current modern approaches are inefficient at fusing multimodal information [76]. The difficulty of fusing features into a single joint representation is one of the primary limitations of early data fusion methods. Moreover, careful data filtering is required to create common ground before fusion. This may pose a problem if the datasets provided are already scarce. Due to the use of different models for each modality, fusing information at the decision level is expensive in terms of learning effort. Therefore, a further layer of learning is necessary for the fused representation. The probable loss of correlation in fused feature space is another limitation [20]. Although a few methods combine them using the attention mechanism, they are not sufficiently fine-grained for the feature fusion [78].

## 6 Future directions

Based on this review, we summarize some of the significant issues in this field that must be addressed to further improve fake news detection techniques.

• Due to the use of either small or imbalanced datasets, detection models suffer from several challenges, including underfitting, overfitting, misclassification, and poor classification, which significantly degrade their performance. The data augmentation method has been widely and successfully applied to image classification, especially using the generative adversarial network (GAN) method. GAN can be used in the field of fake news detection to address these problems and obtain a better training performance of the detection model. Some modifications must be made to the vanilla GAN for it to be applied to the text. This method is effective for addressing the lack of data in the early stages, especially when new events emerge, such as the Covid-19 pandemic.
• Visual features were not widely used in fake news detection. Images in fake news are manipulated in intricate ways to trick viewers, grab their attention, and persuade them to share the news. There is a relationship between textual and visual features that can be employed in fake news detection. Therefore, the combination of text and image features increases detection accuracy.
• Social media language often contains slang or mistakes as social media audiences are composed of different cultures, ages, and educational backgrounds. One of the issues when using embedding models, such as Glove and Word2Vec, is OOV. In order to tackle this problem, models such as BERT and fastText can be used.
• Fake news is often written in capital letters or contains exclamation marks or question marks to grab the reader's attention. Using the normalization method or omitting these two symbols may remove significant features. This is why pre-trained cased embedding models should be utilized, which consider uppercase and lowercase words.
• Each embedding model has its own method of representing features. Therefore, it should take into account the capture of word context in a document, as well as semantic and syntactic similarity, in order to fully exploit the structural features of the text. This leads to increased accuracy of fake news detection.
• Machine-learning methods have been used to detect fake news; however, the accuracy of the detection was observed to be lower. Deep learning algorithms outperformed machine learning algorithms and provided outstanding results in image analysis and text classification, thanks to their ability to handle copious amounts of data, extract features efficiently, learn successfully, and capture complex patterns.
• Concatenating data from different modalities (text and image) may be highly helpful for identifying fake news. However, the real significance of several modalities cannot be determined by directly concatenating the features. The unique features of each modality must be preserved while fusing relevant information between the different modalities. One of the most effective ways to deal with this issue includes the attention mechanism.

## 7 Conclusion

The review article provides a detailed investigation of all types of feature-based methods used in fake news detection models. Studies involving fake news detection were analyzed, and their limitations or weaknesses were highlighted in this study. Based on this analysis of these studies, issues that constitute current challenges to fake news detection models include datasets, feature representation, and data fusion. With the help of the key findings obtained, this review suggests investigating the use of effective techniques to address the main problem in most fake news detection studies, which is either the limited size of the dataset used or its skewed distribution, which leads to several issues, including overfitting and misclassification. Another outcome is the need to take advantage of the visual features and the typical representation of the features in detecting fake news. Furthermore, attention must be paid to common relationships between the different modalities on the one hand, while preserving the unique features of each modality on the other hand, during the integration of features. In addition, a few suggestions have been presented as future directions to address these existing problems. These suggestions will enable future researchers to improve models for detecting fake news. Since studying and addressing the impact of fake news on society is crucial, and taking political factors into account in such studies is essential due to the significant impact that fake news can have on the political sphere, a few recommendations are as follows. Official political speeches and announcements published on social media must be marked with distinctive marks, allowing the public to know the source of this news. Financially independent fact-checking organizations must be supported to verify news content, especially those related to the political aspect, and during election campaigns. Moreover, social media platforms should be urged to combat the spread of fake news, and its promoters and publishers should be punished. The development of news content publishing algorithms that give priority to news from verified sources, provided that it does not conflict with freedom of opinion and expression, may also help in tackling this issue. The comments and replies of the public about the published news can also be used to detect fake news. Social media platforms should be monitored by governments and hold them accountable in the event that they contribute to the spread of false news. Lastly, the academic community should be encouraged and supported for further investigation and development in this field.

## Funding

This research was funded by the 10.13039/501100004515Universiti Kebangsaan Malaysia under Geran Universiti Penyelidikan (GUP), Grant Code: GUP-2020-088.

## Author contribution statement

All authors listed have significantly contributed to the development and the writing of this article.

## Data availability statement

No data was used for the research described in the article.

## Declaration of competing interest

The authors declare that they have no known competing financial interests or personal relationships that could have appeared to influence the work reported in this paper.
